# Competency model for dentists in China: Results of a Delphi study

**DOI:** 10.1371/journal.pone.0194411

**Published:** 2018-03-22

**Authors:** Yunxia Geng, Liying Zhao, Yu Wang, Yiyuan Jiang, Kai Meng, Dongxiang Zheng

**Affiliations:** 1 School of Health Administration and Education, Capital Medical University, Beijing, China; 2 School of Stomatology, Capital Medical University, Beijing, China; National Taiwan University, School of Dentistry, TAIWAN

## Abstract

**Objective:**

With the increasing awareness of the importance of oral health, patients have an increasing need for integrated care from dentists. In China, the dentistry examination consists of two parts: a practical skills examination and a comprehensive medical examination; to date, no assessment methods that are based on specialized dentistry competencies, unlike the United States, Canada, and other countries, have been established. Therefore, the purpose of this study was to construct a competency model for dentists in China in order to guide the development, admission, training and assessment of dentists.

**Methods:**

Using a literature review, focus group interviews and in-depth personal interviews, a dentist competency index was developed with an expert consultation questionnaire. A panel of 20 specialist experts was chosen from ten national medical universities to carry out two rounds of Delphi expert analysis, using the boundary value method to filter the indicators and the Analytic Hierarchy Process to calculate the weights of the primary indicators.

**Results:**

Two rounds of Delphi results showed that the expert authority, enthusiasm, and coordination coefficients were high. Constructs of the competency model that included seven primary indicators and 62 secondary indicators determined the weight of each index. The seven primary indicators included the following: clinical skills and medical services, disease prevention and health promotion, interpersonal communication skills, core values and professionalism, medical knowledge and lifelong learning ability, teamwork ability and scientific research ability.

**Conclusion:**

In conclusion, the use of the Delphi method to construct an initial model of Chinese physician competency is scientific and feasible. The initial competency model conforms to the characteristics and quality requirements of dentists in China and has a strong scientific basis. The dentist competency model should be used in the National Dental Licensing Examination in China.

## Introduction

Oral health is an important part of general health that can affect the quality of life. In 2007, the World Health Organization (WHO) categorized oral disease as a serious public health problem that requires active prevention and treatment. Dentists provide dental technology services to patients, and their clinical competency directly affects the overall level of hospital and patient satisfaction. As the awareness of oral health continues to increase, there is an increasing requirement for dentists to maintain and improve their clinical competency. The training process for dentists in medical and dental schools as well as dentist selection, training, and assessment in dental hospitals has become more stringent. Worldwide and in China, research regarding maintaining clinical competency has become an increasingly important component of medical education and training.

In 1973 at Harvard University, Professor David McClelland proposed the competency concept that includes personal behavioral characteristics that distinguish between levels of achievement and performance in life or work. The concept of competency also proposes that traditional assessments should be replaced with a competency measurement [1].

In 2002, Epstein and Hundert argued that competency for clinicians included the ability to use communication skills expertly and accurately and to use academic knowledge, technical methods, clinical thinking, emotional expression, value orientation and personal experience in daily medical practice in order to benefit individuals and groups [2]. Europe, the US, and developed countries have established medical personnel training programs based on dentist competency, and the training program has become the basis of medical education, training, and assessment [3–6].

The oral practice qualification test is the only way to appoint a dentist. In the five regions of the US, Europe, Canada, Australia, and New Zealand, the dentist competency model has the same components: clinical practice skills, oral-related clinical expertise, professional attitudes and professional ethics, interpersonal communication and teamwork. However, the content of the competency model still has some differences in the five regions.

At present, in China, a dental student undertakes five years of undergraduate education, followed by a postgraduate year of standardized training (known as a "5 + 1" stage dentist in this paper), before applying for the National Licensing Examination. However, China has not yet established job competency-oriented assessment criteria despite some research in this area and has not yet built a dentist competency model. Therefore, this study was undertaken to use the Delphi method to construct a competency model for dentists in China.

## Materials and methods

### Ethics statement

Ethics approval was granted by the Beijing Dental Hospital Research and Ethics Committee. The study design and information sheets were reviewed by the committee and considered appropriate for use. All 20 experts signed informed consents and voluntarily participated in the study. Participant information was confidential, and participants were able to withdraw at any time during the study.

### Use of the Delphi model

Delphi was originally developed by the Rand Corporation. Some experts observe that when researchers lack scientific knowledge of the topic being investigated or face-to-face data collection is impractical, the Delphi method can be applied[7,8]. In this study, we used the Delphi expert consultation method, the threshold method and the analytic hierarchy process (AHP) to establish the initial model for "5 + 1" stage dentist competency [9, 10].

#### Preliminary competency indicators

First, the framework of the Chinese University Hospitals’ general model for clinicians was used for reference [11]. Second, a literature search and review were done using databases that included Wan Fang, CNKI, and PubMed to study the models of dentist competency from Canada, Europe, New Zealand, the USA and Australia. Third, six focus group interviews were performed. Finally, the initial physician competency model was constructed. The model included eight first-level indices and 81 second-level indices, resulting in a "dentist competency Delphi questionnaire."

#### Design of the Delphi consulting questionnaire

The expert consultation questionnaire included the background, purpose, and expert basic information questionnaire, AHP matrix table, index table of all levels, degree of expertise quantification table, degree of expert opinion coordination and quantification table, and opinion column.

The index evaluation included three aspects: importance, feasibility, and sensitivity. The scores were between 1–10, where 1 means that the index is least important, least feasible, and least sensitive and 10 means that the index is most important, most feasible, and most sensitive. At the same time, experts needed to select the familiarity and judgment scores. Familiarity was divided into a scale of 1–5 using the Likert scale method, where 1 means that the expert is most unfamiliar with the index and 5 means that the expert is most familiar with the index. Judgment was based on four aspects: theoretical analysis, work experience, understanding of domestic and foreign counterparts, and insight. The judgment scores were used to determine the degree of influence, with scores of 1–3 points, where 3 = high, 2 = medium, 1 = small.

#### Selection of the experts

There are 20 experts in 10 national dentistry qualification examination areas, from 10 universities of stomatology and their affiliated dental hospitals: the Fourth Military Medical University, Lanzhou University, Sichuan University, Jilin University, Nanjing Medical University, Shanghai Jiao Tong University, Capital Medical University, Wuhan University, Sun Yet-sen University and Chongqing Medical University.

The specialties of the experts included management, education, and dentistry. All experts in their respective fields had high academic attainments; 20 experts had master's degrees or higher, and 75% had a doctorate or higher education. Information regarding the experts is shown in Table 1.

**Table 1:** Participant information.

	Participants	Number	Percent
**Qualification**	Master’s	5	25
	Ph.D.	15	75
**Professional Title**	Middle	2	10
	High	12	60
	Senior	6	30
**Working years**	<5	0	0
	6–10	5	25
	11–15	6	30
	16–20	2	10
	>20	7	35
**Profession**	Journal of Oral and Maxillofacial Surgery	4	20
	Department of Oral Medicine	2	10
	Department of Orthodontics	1	5
	Department of Dental Pulp	3	15
	Department of Pathology	1	5
	Hospital management	1	5
	Scientific research and teaching	4	20
	Comprehensive department	4	20

#### Implementing two rounds of Delphi expert advice

Two rounds of Delphi expert advice were implemented in writing to the 20 experts who were asked to evaluate each indicator and make comments. After the first round of the survey, SPSS 16.0 statistical software was used to analyze the questionnaire responses. The degree of coordination of the experts was calculated to include the experts’ positive coefficient and degree of authority according to the boundary value method to reduce the competency index. The indicators were adjusted and supplemented according to the comments of the experts. A second round of expert consultation was made followed by further analysis of the questionnaire and revision of the competency index and calculation of the index. Finally, the competence model for dentists was constructed.

### Data analysis

#### Positive coefficient of experts

The positive coefficient of experts is the effective recovery rate of the expert consultation questionnaire and can reflect positive input from the experts. The American sociologist Earl Babble argued that a 50% recovery rate was the minimum acceptable rate for analysis and reporting, 60% could be considered good, and 70% achieved a very good standard [12].

#### Degree of expert authority

The degree of expert authority was determined by two factors: the expertise to make a judgment on the program, and the familiarity of the expert with the problem [13]. In this study, Ca represents the coefficient that affects the expert judgment. The experts used the terms "practical experience," "theoretical analysis," "understanding of peers," and "insight" as the basis for judgments. Large, medium, and small judgments were made to determine the extent of influence. When Ca = 1, the degree of influence of expert judgment is medium, and when Ca = 0, it has no effect on expert judgment according to the valuation criteria in Table 2.

**Table 2:** Judgment basis and influence degree.

Judgments based	Degree of influence		
	Small(0)	Middle(0.5)	High(1)
Working Experience (0.4)	0	0.2	0.4
Theoretical analysis (0.3)	0	0.15	0.3
Peer understanding (0.2)	0	0.1	0.2
Intuition (0.1)	0	0.05	0.1
Total	0	0.5	1

Familiarity was expressed in terms of Cs. This study used the Likert scale method to classify experts into five levels of familiarity: very familiar (5 points), more familiar (4 points), generally familiar (3 points), less familiar (2 points), and not familiar (1 point) (Table 3). The degree of familiarity of each expert was calculated statistically.

**Table 3:** Degree of familiarity of expert knowledge.

Familiarity	Coefficient
Very familiar	1
More familiar	0.75
Generally familiar	0.5
Less familiar	0.25
Unfamiliar	0

The degree of expert authority was expressed by Cr: Cr = (Ca + Cs) / 2; values greater than 0.7 were considered to be acceptable [14].

#### Degree of expert coordination

The degree of expert coordination is an important index for judging the consistency of the indicators among the experts, and included the Kendall W coordination coefficient and the coefficient of variation of each index; the coefficient of variation is an important basis for index deletion [15]. The smaller the coefficient of variation, the greater is the degree of coordination of the experts.

#### Boundary value calculation methods and results

The method of calculating the cut-off value of the full frequency and arithmetic mean was as follows: the mean value = mean—standard deviation; a score higher than the cut-off value was chosen. The calculation results of the boundary values are shown in Table 4.

**Table 4:** Threshold table.

	Importance			Feasibility			Sensitivity		
	M	S	D	M	S	D	M	S	D
Full frequency	0.42	0.17	0.25	0.23	0.17	0.06	0.23	0.14	0.08
Mean	8.62	0.59	8.03	7.64	0.78	6.86	7.3	0.78	6.52
Coefficient of variation	0.17	0.06	0.22	0.25	0.07	0.32	0.33	0.09	0.41

**Note: M** is an abbreviation for **Mean**

**S** is an abbreviation for **Standard**

**D** is an abbreviation for **Deviation**

#### Guidelines for the selection of indicators

There are three indicators, where the first is the importance of the three indicators does not meet the requirements; the second is two boundaries of the importance, feasibility, and sensitivity do not meet the requirements; and the last one is deleting or modifying the indicator according to the expert opinion.

#### Index weight calculation method

In order to reflect the importance of the dentistry competency index, another AHP software was used to calculate the weight of the primary index in the second round. The weight of the secondary index was calculated by the percentage weight method. In this study, we used the scale matrix judgment method to allow each of the experts to compare each indicator in each row and column and to complete the results in the corresponding form.

## Results

### Positive coefficient of experts

In the first round of Delphi expert consultation, 20 questionnaires were sent out, and 20 were effectively recovered, resulting in an effective recovery rate of 100%. In the second round, 20 questionnaires were issued, and the effective recovery rate was also 100%. Some experts modified and improved a number of indicators.

### Expert authority

The expert authority coefficients were 0.74 and 0.75 in two rounds of consultation; both of these were greater than 0.7, indicating that the expert consultation results were accurate and credible (Table 5).

**Table 5:** Coefficients of expert authority.

Primary indicator	First round			Second round		
	Ca	Cs	Cr	Ca	Cs	Cr
Clinical Skills and Medical Services	0.75	0.89	0.82	0.73	0.95	0.84
Disease Prevention and Health Promotion	0.71	0.85	0.78	0.72	0.9	0.81
Information Collection and Management Capability	0.59	0.68	0.63	0.63	0.69	0.66
Medical Knowledge and Lifelong Learning Ability	0.73	0.79	0.76	0.67	0.84	0.75
Interpersonal Communication Skills	0.66	0.83	0.74	0.64	0.85	0.74
Teamwork	0.61	0.8	0.71	0.61	0.83	0.72
Scientific Research Ability	0.69	0.79	0.74	0.62	0.86	0.74
Core Values and Professional Qualities of Doctors	0.59	0.84	0.72	0.65	0.86	0.75
Mean	0.67	0.81	0.74	0.66	0.85	0.75

### Expert consultation coordination

The P-value of the coordination coefficient was less than 0.01 (Table 6), indicating that the expert opinion was consistent.

**Table 6:** Coordination factors of expert consultations.

Round	Indicator level	Importance			Feasibility			Sensitivity		
		W	χ2	P	W	χ2	P	W	χ2	P
**First**	Primary indicator	0.47	62.9	<0.01	0.33	41.2	<0.01	0.44	57.99	<0.01
	Secondary indicator	0.19	270.1	<0.01	0.23	326	<0.01	0.17	231.1	<0.01
**Second**	Secondary indicator	0.23	317	<0.01	0.22	304	<0.01	0.2	262.4	<0.01

### Index screening

#### Screening results of the primary indicator

Statistical analysis of the experts’ scores after the first round of consultation showed that the boundaries of "information and management capacity" and "scientific research capacity" did not meet the study requirements (Table 7). The results of the second round of expert consultation showed that the "information and management capacity" had the lowest weight (0.0668), followed by "scientific research" (0.849). Finally, combined with the views of the experts, the decision was made to delete "information and management capabilities" and retain "scientific research capabilities."

**Table 7:** Filtered results of primary indicators.

Primary indicator	Importance			Feasibility			Sensitivity			Results
	X	CV	F	X	CV	F	X	CV	F	
Clinical Skills and Medical Services	9.80	0.05	0.85	9.15	0.11	0.50	9.30	0.11	0.60	
Disease Prevention and Health Promotion	8.35	0.20	0.35	7.35	0.24	0.10	7.05	0.25	0.10	
Information Collection and Management Capability	**6.90**	**0.28**	**0.10**	**6.55**	0.31	0.10	**6.20**	0.30	**0.05**	Delete
Medical Knowledge and Lifelong Learning Ability	8.70	0.13	0.35	6.55	0.29	0.05	7.20	0.27	0.15	
Interpersonal Communication Skills	8.70	0.13	0.30	7.30	0.26	0.15	7.65	0.25	0.20	
Teamwork	8.15	0.17	0.25	6.55	0.34	0.10	7.15	0.27	0.05	
Scientific Research Ability	**7.20**	0.17	**0.00**	**6.85**	**0.33**	0.20	**6.20**	0.31	**0.00**	Reserved
Core Values and Professional Qualities of Doctors	8.40	0.17	0.25	6.90	0.27	0.10	7.50	0.29	0.15	

Note: the bold value did not meet the boundary value, F on behalf of the full frequency

#### Screening results of the secondary indicator

Using the boundary value method combined with expert advice in the first round, we deleted a total of 10 secondary indicators. In the second round, we deleted nine secondary indicators (Table 8). Additionally, four secondary indicators were revised after considering expert advice (Table 9).

**Table 8:** Secondary indicators that were deleted in two rounds of Delphi.

Secondary index	Importance			Feasibility			Sensitivity		
	X	CV	F	X	CV	F	X	CV	F
1.12 Can complete a specific workload.**	**7.95**	0.21	**0.25**	8.15	**0.26**	0.40	**7.75**	**0.32**	0.35
1.19 Can identify the psychological and social factors of oral disease in patients and properly deal with the adverse effects of psychological and behavioral factors.**	8.85	0.16	0.50	**7.45**	**0.27**	**0.15**	**7.20**	**0.32**	**0.10**
2.3 Understand their duties, to cooperate with the management of health systems.**	**7.70**	**0.24**	**0.25**	**7.20**	**0.31**	**0.10**	**7.00**	**0.35**	**0.15**
2.4 Understand the structure and function of the health system.*	**7.68**	**0.27**	0.32	7.37	0.25	0.11	6.89	0.36	**0.05**
2.8 Objectively assess the short-term and long-term effects of oral health strategies.**	**8.25**	**0.23**	0.35	**7.10**	0.28	0.10	**6.85**	**0.34**	**0.10**
3.2 Can use information technology effectively to communicate with doctors, nurses and mechanics and educate patients with health knowledge.**	**7.90**	**0.23**	**0.30**	7.80	0.21	0.15	**6.90**	0.31	**0.15**
3.3 Reasonably control the patient's medical expenses.**	**8.25**	0.19	**0.30**	**7.55**	0.28	0.20	**7.20**	**0.33**	**0.15**
3.4 Effectively arrange their own work and do a good job of career planning. *	**7.89**	**0.27**	0.32	6.89	0.27	**0.05**	**6.47**	0.40	0.11
3.5 Can be well self-managed and deal with their own activities reasonably. **	**8.00**	0.20	**0.21**	**6.74**	0.27	**0.05**	6.53	**0.44**	0.11
3.6 Continuously improve organizational coherence and leadership in medical practice.*	**7.32**	**0.28**	**0.21**	**6.26**	**0.36**	**0.00**	**6.00**	**0.42**	**0.00**
3.10 Can use modern information technology to conduct a reasonable publicization of the self.*	**7.20**	**0.29**	**0.15**	7.00	0.28	**0.05**	6.30	**0.42**	0.10
5.8 Euphemistically conveys negative news to patients.**	8.65	0.15	0.40	7.95	0.21	0.20	7.55	0.26	**0.15**
6.3 Caring for colleagues, willing to help colleagues.*	8.15	0.22	0.30	**6.75**	**0.35**	0.10	**6.00**	**0.50**	0.15
6.4 Understand the roles and responsibilities of others on the team.*	**7.95**	0.21	0.25	**6.85**	**0.33**	0.15	**6.25**	**0.42**	**0.05**
7.4 Can raise questions and assumptions and consciously develop their own creative thinking and innovation.**	**8.10**	**0.21**	**0.30**	**7.45**	**0.29**	0.20	**7.25**	0.30	0.20
7.6 Write and publish research articles actively.*	**7.20**	**0.29**	**0.15**	7.35	0.32	0.20	6.65	0.34	0.10
8.1 Adhere to the principle during their career: serve the patient and care about the patient's health.**	8.55	0.19	0.40	**7.45**	**0.28**	0.20	7.50	0.31	0.20
8.2 Cultivating core values, including altruism, pursuing excellence and indifferent to fame and fortune.*	8.25	0.18	0.30	**6.40**	0.31	**0.05**	**6.50**	0.41	0.10
8.8 Fairly and reasonably use medical services resources.*	8.68	0.16	0.42	**6.74**	0.33	**0.05**	**6.42**	**0.47**	0.11

Note

* means: deleted indicators in the first round.

** means: deleted indicators in the second round.

bold values mean it does not meet the standard.

**Table 9:** Indicator changes in two rounds of Delphi.

	Original indicators	Modified results
First round	Master oral surgery for local anesthesia and treatment-related complications.	Modified to: master the oral surgery local anesthesia technology
Second round	Rational use of medical and health resources.	Modified to: in the course of treatment, reasonable use of pharmaceutical supplies, to avoid unnecessary waste.
	Keep an accurate, consistent, and clear patient management record, including referral, commission or transfer of records.	Modified to: continuously, accurately and clearly record the patient management process, including referral, commission or transfer of records.
	Have the ability to read the literature, to carry out an academic literature review, and application and dissemination of knowledge.	Modified to: with the literature reading ability, can carry out an academic literature review.

#### Index system and weighting results

Using the analytic hierarchy process and the percent weight method to determine the weight of the primary and secondary indicators, obtaining the weight of all indicators resulted in the development of the China dentist competency model (Table 10).

**Table 10:** Dentist competency model.

Primary indicator	Secondary indicator	Weight
Clinical skills and medical services(0.2410)	1.1 Complete and accurate collection of important medical history.	0.0135
	1.2 Standard medical record writing ability.	0.0135
	1.3 Relatively standard for oral-related physical examination.	0.0134
	1.4 Proper use of commonly used equipment and supplies and able to standardize the basic oral treatment operations.	0.0133
	1.5 Independent reception capacity.	0.0131
	1.6 Ability to combine theoretical knowledge with clinical practice.	0.0131
	1.7 Translate the terminology into language that is easy for the patient to understand. Develop and discuss treatment plans, cost estimates, time requirements, and patient responsibilities.	0.0129
	1.8 Correctly select the auxiliary inspection items.	0.0129
	1.9 Mastering oral surgery for local anesthesia and treatment-related complications.	0.0127
	1.10 Report orally to a superior doctor the standard clinical problems encountered and analysis of the explanation.	0.0126
	1.11 Explicit indications and contraindications of medications used in oral procedures and correct prescription of medications for oral treatment.	0.0126
	1.12 Master the skills of mainstream technology.	0.0125
	1.13 In the course of oral therapy, consider the patient's needs, explain the etiology, diagnosis, treatment results, risks, benefits and the expected effect of different treatment programs, grasp the overall goal of oral care.	0.0125
	1.14 Can identify and actively participate in the general, acute, heavy, dangerous patients in the field treatment.	0.0124
	1.15 Multi-disciplinary comprehensive analysis capabilities.	0.0124
	1.16 Process of patient referral, including referral, referral or transfer records, can be recorded consistently, accurately and clearly.	0.0122
	1.17 Use evidence-based medicine to make health care decisions, and use a reasonable diagnosis and treatment plan.	0.0120
	1.18 For difficult cases, have a certain degree of independent analysis	0.0119
	1.19 Reasonable and effective management of patients.	0.0116
Disease prevention and health promotion(0.1632)	2.1 Find statutory infectious diseases and report them in a timely manner.	0.0290
	2.2 For the prevention and treatment of oral diseases.	0.0286
	2.3 Prevent the spread of infectious diseases by following current infection control guidelines.	0.0280
	2.4 Recognize oral health for the individual and the important role of the health of the population and actively participate in oral health education and health promotion.	0.0272
	2.5 Assess the patient's oral disease or risk factors for injury.	0.0267
	2.6 In the diagnosis and treatment of rational use of medical supplies, avoid unnecessary waste.	0.0238
Interpersonal communication skills(0.1402)	3.1 To protect patients’ right to know, access to informed consent of patients.	0.0146
	3.2 Protection of patient privacy.	0.0145
	3.3 Attentively listen, collect information related to synthesis and patient issues.	0.0142
	3.4 Understand, trust and respect patients and their families.	0.0141
	3.5 Actively prevent and resolve doctor-patient conflicts.	0.0141
	3.6 Effective oral expression and transmission of information capabilities.	0.0140
	3.7 Appease the patient's anger and misunderstanding mood.	0.0139
	3.8 With the patients and their families, make clinical decisions.	0.0138
	3.9 Communicate effectively with patients, parents or guardians, employees, colleagues, other health professionals and the public.	0.0138
	3.10 Properly deal with the ethical issues arising in the health care process.	0.0132
Core Values and Professional Qualities of Doctors(0.1309)	4.1 Have occupational health and occupational protection awareness; reduce the oral process of occupational hazards.	0.0122
	4.2 With the protection of peer awareness, have respect for peer review and treatment advice and recommendations.	0.0122
	4.3 With the idea of love and compassion, safeguard patients’ rights, privacy and interests.	0.0122
	4.4 Sincere and trustworthy, strong sense of responsibility, with a positive attitude and professionalism.	0.0121
	4.5 Physical and mental health, with patience and endurance, has a good psychological adjustment and compression capacity, maintain self-care ability.	0.0121
	4.6 In potential medical disputes, have early warning consciousness	0.0121
	4.7 Ability to assess the clinical expertise of the individual, to recognize the limitations of the individual, and to know when to consult or seek advice.	0.0120
	4.8 Have a contingent capacity and response to emergency.	0.0119
	4.9 With the identity of the doctor's makeup.	0.0114
	4.10 Show self-discipline and patient-centeredness in order to maximize achieving the interests of patients.	0.0114
	4.11 Have rigorous, meticulous, and keen insight.	0.0113
Medical Knowledge and Lifelong Learning Ability(0.1246)	5.1 Master and apply the basic knowledge of clinical medicine.	0.0220
	5.2 Practice concerns, including new materials and new technologies, including oral dynamic cutting-edge, constantly updated knowledge and professional skills	0.0215
	5.3 Have basic biomedical knowledge.	0.0212
	5.4 Actively participate in continuing education.	0.0212
	5.5 Knowledge of behavioral and social sciences, medical ethics and law.	0.0196
	5.6 Have certain professional foreign language skills.	0.0191
Teamwork(0.1116)	6.1 Follow the doctor's orders.	0.0232
	6.2 Be able to work with colleagues and respect their abilities and contributions.	0.0226
	6.3 Can establish good cooperative relationship with other teams.	0.0223
	6.4 Good coordination with team members to avoid conflict.	0.0219
	6.5 Develop a patient-care plan in a team-based manner	0.0216
Scientific research ability(0.0886)	7.1 Have critical thinking skills in professional activities and make appropriate medical decisions.	0.0184
	7.2 Understand the complexity and uncertainty of health care activities.	0.0181
	7.3 Have the ability to read the literature; can carry out academic literature review.	0.0176
	7.4 Use different databases and other means to retrieve, collect, and analyze relevant medical information.	0.0174
	7.5 Actively participate in research activities in this field.	0.0172

## Discussion

In recent years, the Delphi method has been widely used in health management and has become a mature indicator screening method in the field of health care [16–20]. This method combines qualitative and quantitative research methods to collect and screen expert advice. In the consultation process, the choice of experts is a key link. Inappropriate selection of expert members will not only increase the bias in the evaluation but may also lead to a decrease in the response rate. In general, there is a functional relationship between the accuracy of the consultation results and the number of participants in the consultation; between 15 and 50 experts is considered to be suitable [21]. Based on the principle of a combination of representation and authority, this study selected 20 experts and scholars involved in the training of dentists in 10 universities nationwide in China. The authority of the experts was greater than 0.7, and the chi-squared test results of the coordination coefficient showed that the P-values were less than 0.01. In the first round, the coefficient of variation was 0.05–0.50, and in the second round, it was 0.04–0.35, indicating that the participation of the experts was focused and detailed. Two rounds of expert consultation questionnaires had an effective recovery rate of 100%, showing that the experts were motivated and enthusiastic to participate.

In the index screening, we selected three indicators for the screening items: full frequency, arithmetic mean, and coefficient of variation. Each project determined the threshold. From the analysis of the screening results, they appeared to be reliable; the weight value of the dentist competency index reflected the importance of the indicator. Weight is the importance of indicators and the size of the quantitative performance; a reasonable weight setting is significant for establishing an index system. In order to quantify the importance of competency, we used the analytic hierarchy process to calculate the weight of the first-level index, used the experts’ importance score, and combined the weight of the primary index to calculate the weight of the secondary index, so that the calculation of the weight of the evaluation index was more scientific.

In this study, among the first-level indicators, the top three weights were clinical skills and medical services (0.2309), disease prevention and health promotion (0.1564), and interpersonal communication skills (0.1343); these weights were related to the occupational characteristics and competence of the dentists. Dental practitioners are knowledge-based skilled personnel with solid professional theoretical knowledge and skill. In addition to a high level of practical skills, each dentist must be able to ensure harmonious dentist-patient relationships; these relationships require effective communication between dentists and patients, as well as good interpersonal and communication skills. Also, because prevention is becoming an increasing part of clinical practice, the dentist has a role in patient and community health education.

In this study, the weights of "scientific research ability" and "core values and professionalism" were analyzed through two rounds of expert consultation. We merged the second index of "information and management ability" into the "scientific research ability" index. The subjects of the study were dentists who had graduated from medical and dental colleges and universities and received a one-year standardized training in residency. The main goal for dentists at this stage is clinical skills training. From the dental student’s point of view, the vast majority of students also believe that practical clinical teaching is helpful when supported by clinical research. Experts also believe that dental training should be based on clinical skills; some controversy regarding including research in the training process remains.

In the treatment of patients, the dentist usually works independently. Because of the limited number of nurses, China's "four-hand" operation is not universal. Therefore, in this study, the "team cooperation ability" index weight value was low. In the interview, some experts noted that the practice of the dentist was uniquely independent and that the dentist’s competency and the clinical physician’s competency have some differences.

According to the competency “onion model,” among the seven first-level indicators we obtained in this study, "clinical skills and medical services" and "disease prevention and health promotion" reflect knowledge and skills; "medical knowledge and learning ability," "interpersonal communication skills," "teamwork ability," and "scientific research ability" reflect self-awareness and training ability; and "core values and professionalism" reflect characteristics and motives.

There are 62 second-level indicators in the dentist competency model; this number is less than the 82 second-level indicators in the competency training for physicians and surgeons in China[11]. Because the dentists in this study had standardized training for one year after graduating from university, and the physicians and surgeons had standardized graduate training for three years after graduating from college, there are differences in the competency indicators. Furthermore, this study reflects the professional characteristics of dentistry and includes the evaluation of practical skills, such as tooth repair, surgical extraction, and traditional preparation of dentures, as well as management abilities including the rational use of drugs, avoiding unnecessary waste, and local anesthesia technology skills. Dentists also need to inform patients about oral health and actively participate in oral health education and promotion.

Compared with the competency model of the other five countries, we found the following commonalities and differences. We all have the following four competencies: “Professionalism”, “Communication and Interpersonal Skills”, “Health Promotion”, and “Practice Management and Informatics”. However, “Core Values and Professional Qualities of Doctors” is also important in this study and helps define the first competencies. In China, we place great emphasis on the cultivation of doctors' values and their enthusiasm for work. In addition, each of the competencies has a corresponding weight that helps us to apply our model to practical work, including the development, admission, training and assessment of dentists.

This study had several limitations. The use of a single model, the Delphi model, to construct the competency model for dentists in China was not verified with the use of alternative models. The constructed model has not yet been tested in clinical practice or postgraduate dental training and evaluation. Although the experts in the study had discipline, enthusiasm, and national geographical representation, their input was subjective and not objective [22]. Therefore, future follow-up studies on the use of the model will evaluate and validate the model with a behavioral event interview method in addition to being assessed nationwide in China to develop a dentist competency model that is suitable for our country.

## Conclusion

This study describes the process of constructing the initial model of dentist competency using the Delphi method to develop an assessment method that contains seven primary indicators and 62 secondary indicators. The dentist competency model is scientifically based and clinically relevant and should be suggested for use in the National Dental Licensing Examination in China.

## Supporting information

S1 File Expert consultation questionnaire (first round). (DOC)

S2 File Expert consultation questionnaire (second round). (DOC)

S3 File Expert consultation questionnaire (first round) in Chinese. (DOC)

S4 File Expert consultation questionnaire (second round) in Chinese. (DOC)
