# Supplementary material for: Competency model for dentists in China: Results of a Delphi study
Source: PLoS One. 2018 Mar 22;13(3):e0194411. doi: 10.1371/journal.pone.0194411 (PMC5864007; doi:10.1371/journal.pone.0194411)
Supplement: S1 File — (DOC) [file pone.0194411.s001.doc]

**Expert Consultation Questionnaire of Dentist Competency**

**Model Study(first round)**

Dear experts:

Thank you for participating in expert advice! In order to know more about our subject, please read the relevant contents of this study.

We assume the task entrusted by the National Center for Medical Examination to study the issue of "Competence Model of Dentist ". This study aims to summarize the competence of "5 +1" stage dentist （who completed a one-year probationary period after graduating from dental specialty and to prepare for oral qualification examination）through expert consultation and Delphi method. Then build a competency index system. Initially, the index system divided into two levels. There are eight first level indicator: Clinical skills and medical services , Disease prevention and health promotion, Information and management skills, Medical knowledge and lifelong learning ability, Interpersonal communication skills, Teamwork skills, Scientific research ability, Core values ​​and professionalism of doctors. Secondary indicators in the further development of an indicator. We need to determine the weighting factor for each secondary indicator. Finally, a scientifically reasonable and operable index system is formed, so as to construct a dentist competency model that suits our national conditions. The research results of the subject can provide the research basis for the introduction of the concept of "competency" in the qualification examination of dentists and provide a more scientific and quantitative basis for the training, selection, training and assessment of stomatology.

To save time and ensure the progress of research, we use email to conduct two rounds of consultation. Please fill in the questionnaire item by item to avoid omission. Your selection and scoring is of the utmost importance to us. Please complete and send e-mail to:kqyssrl@sina.com within one week. If you have questions about the questionnaire, please call or send an e-mail consultation. We will keep the confidentiality of your completed questionnaire and sincerely thank you for your support for this research.

Contact:

Kai Meng 13261365511 Dongxiang Zheng 13681036988

Beijing Stomatological Hospital of Capital Medical University

**A Detailed Explanation of the Criteria For Selecting Indicators**

1 the Importance of Indicators: 1 is the most important, 10 is the most important

2 the Feasibility of Indicators: Feasibility is the difficulty and cost of obtaining the indicator in the actual evaluation. The easier it is to obtain the indicator, the more viable it is. 1 point is the most impractical indicator, 10 points is the most viable indicator

3 the sensitivity of indicators: Sensitivity refers to whether indicators can better reflect the "5 + 1" stage dentist's competence. If the sensitivity of an indicator is high, it shows that the improvement of this indicator can greatly enhance the degree of reflection on the competence of dentists.10 points is the indicator has the strongest sensitivity

4 The research design of the index system is more, which index do you think is not important or not easy to obtain (not feasible), please mark the "×" behind the indicator, if you want to increase the indicator, then fill in the suggestion column below and score.

5 Your judgment on the index is affected by four factors: 1Working Experience 2Theoretical analysis 3Peer understanding 4Intuition .The influence degree is divided into three levels. Please according to your judgment of each index to choose the appropriate level of influence.

6 Your understanding of the index is divided into five levels; " 5 very familiar 4 more familiar3 general2 less familiar1 1 unfamiliar" Please according to the actual situation fill the score in the table.

**Personal information**

Name Employer

Phone number Emai

Professional Title

Qualification ①Undergraduate ②Masters ③Ph.D

Working years ①≤5 ②6—10 ③11—15 ④16—20 ⑤>20

Profession ①Journal of Oral and Maxillofacial Surgery ②Department of Oral Medicine

③Department ofOrthodontics ④Department of Dental Pulp ⑤Department of Pathology ⑥Hospital management ⑦Scientific research and teaching

⑧Comprehensive department

| **Table 1: Primary indicator evaluation table** | | | | | | | | | | |  |
| --- | --- | --- | --- | --- | --- | --- | --- | --- | --- | --- | --- |
|  | **Primary indicator** | importance  （1-10） | feasibility  （1-10） | Sensitivity  （1-10） | Familiarity:  5 = Very familiar  4 = More familiar  3 = General  2 = Less familiar  1 = Unfamiliar | Judgment basis and degree of influence (degree of influence divided into three levels 3 = large, 2 = medium, 1 = small) | | | | modify | |
|  |  |  |  |  |  | Working Experience | Theoretical analysis | Peer understanding | Intuition |  |  |
| 1 | Clinical Skills and Medical Services |  |  |  |  |  |  |  |  |  | |
| 2 | Disease Prevention and Health Promotion |  |  |  |  |  |  |  |  |  | |
| 3 | Information Collection and Management Capability |  |  |  |  |  |  |  |  |  | |
| 4 | Medical Knowledge and Lifelong Learning Ability |  |  |  |  |  |  |  |  |  | |
| 5 | Interpersonal Communication Skills |  |  |  |  |  |  |  |  |  | |
| 6 | Teamwork |  |  |  |  |  |  |  |  |  | |
| 7 | Scientific Research Ability |  |  |  |  |  |  |  |  |  | |
| 8 | Core Values and Professional Qualities of Doctors |  |  |  |  |  |  |  |  |  | |
| Modify: | | | | | | | | | | | |

**Table 2: Secondary index system evaluation table**

| Primary indicator |  | Secondary index | importance  （1-10） | feasibility  （1-10） | Sensitivity  （1-10） | Familiarity:  5 = Very familiar  4 = More familiar  3 = General  2 = Less familiar  1 = Unfamiliar | Judgment basis and degree of influence (degree of influence divided into three levels 3 = large, 2 = medium, 1 = small) | | | | | modify |  |
| --- | --- | --- | --- | --- | --- | --- | --- | --- | --- | --- | --- | --- | --- |
|  |  |  |  |  |  |  | Working Experience | Theoretical analysis | Peer understanding | | Intuition |  |  |
| Clinical Skills and Medical Services | 1.1 | Complete and accurate collection of important medical history. |  |  |  |  |  |  |  | |  |  |  |
|  | 1.2 | Relatively standard for oral-related physical examination. |  |  |  |  |  |  |  | |  |  |  |
|  | 1.3 | Correctly select the auxiliary inspection items. |  |  |  |  |  |  |  | |  |  |  |
|  | 1.4 | Report orally to a superior doctor the standard clinical problems encountered and analysis of the explanation. |  |  |  |  |  |  |  | |  |  |  |
|  | 1.5 | Use evidence-based medicine to make health care decisions, and use a reasonable diagnosis and treatment plan. |  |  |  |  |  |  |  | |  |  |  |
| **Primary indicator** |  | Second index | importance  （1-10） | feasibility  （1-10） | Sensitivity  （1-10） | Familiarity:  5 = Very familiar  4 = More familiar  3 = General  2 = Less familiar  1 = Unfamiliar | Judgment basis and degree of influence (degree of influence divided into three levels 3 = large, 2 = medium, 1 = small) | | | | | modify |  |
|  |  |  |  |  |  |  | Working Experience | Theoretical analysis | Peer understanding | | Intuition |  |  |
| Clinical Skills and Medical Services | 1.6 | Can identify and actively participate in the general, acute, heavy, dangerous patients in the field treatment. |  |  |  |  |  |  |  | |  |  |  |
|  | 1.7 | Independent reception capacity. |  |  |  |  |  |  |  | |  |  |  |
|  | 1.8 | Multi-disciplinary comprehensive analysis capabilities. |  |  |  |  |  |  |  | |  |  |  |
|  | 1.9 | The ability to combine theoretical knowledge with clinical practice. |  |  |  |  |  |  |  | |  |  |  |
|  | 1.10 | Proper use of commonly used equipment and supplies and able to standardize the basic oral treatment operations. |  |  |  |  |  |  |  | |  |  |  |
|  | 1.11 | Standard medical record writing ability. |  |  |  |  |  |  |  | |  |  |  |
|  | 1.12 | Can complete a specific workload. |  |  |  |  |  |  |  | |  |  |  |
| **Primary indicator** |  | Second index | importance  （1-10） | feasibility  （1-10） | Sensitivity  （1-10） | Familiarity:  5 = Very familiar  4 = More familiar  3 = General  2 = Less familiar  1 = Unfamiliar | judgment basis and degree of influence (degree of influence divided into three levels 3 = large, 2 = medium, 1 = small) | | | | | modify |  |
|  |  |  |  |  |  |  | Working Experience | Theoretical analysis | Peer understanding | | Intuition |  |  |
| Clinical Skills and Medical Services | 1.13 | For difficult cases have a certain degree of independent analysis. |  |  |  |  |  |  |  | |  |  |  |
|  | 1.14 | Master the skills of mainstream technology. |  |  |  |  |  |  |  | |  |  |  |
|  | 1.15 | In the course of oral therapy, consider the patient's needs, explain the etiology, diagnosis, treatment results, risks, benefits and the expected effect of different treatment programs, grasp the overall goal of oral care. |  |  |  |  |  |  |  | |  |  |  |
|  | 1.16 | Translate the terminology into language that is easy for the patient to understand. Develop and discuss treatment plans, cost estimates, time requirements, and patient responsibilities. |  |  |  |  |  |  |  | |  |  |  |
| **Primary indicator** |  | Second index | importance  （1-10） | feasibility  （1-10） | Sensitivity  （1-10） | Familiarity:  5 = Very familiar  4 = More familiar  3 = General  2 = Less familiar  1 = Unfamiliar | Judgment basis and degree of influence (degree of influence divided into three levels 3 = large, 2 = medium, 1 = small) | | | | | modify |  |
|  |  |  |  |  |  |  | Working Experience | Theoretical analysis | Peer understanding | | Intuition |  |  |
| Clinical Skills and Medical Services | 1.17 | Master oral surgery for local anesthesia and treatment-related complications. |  |  |  |  |  |  |  | |  |  |  |
|  | 1.18 | Explicit indications and contraindications of medications used in oral procedures, and correct prescription of medications for oral treatment. |  |  |  |  |  |  |  | |  |  |  |
|  | 1.19 | Can identify the psychological and social factors of oral disease in patients, and properly deal with the adverse effects of psychological and behavioral factors. |  |  |  |  |  |  |  | |  |  |  |
| Disease Prevention and Health Promotion | 2.1 | Found and timely reported to the statutory infectious diseases. |  |  |  |  |  |  |  | |  |  |  |
|  | 2.2 | For the prevention and treatment of oral diseases. |  |  |  |  |  |  |  | |  |  |  |
| **Primary indicator** |  | Second index | importance  （1-10） | feasibility  （1-10） | Sensitivity  （1-10） | Familiarity:  5 = Very familiar  4 = More familiar  3 = General  2 = Less familiar  1 = Unfamiliar | Judgment basis and degree of influence (degree of influence divided into three levels 3 = large, 2 = medium, 1 = small) | | | | | modify |  |
|  |  |  |  |  |  |  | Working Experience | Theoretical analysis | Peer understanding | | Intuition |  |  |
| Disease Prevention and Health Promotion | 2.3 | Understand their duties, to cooperate with the management of health systems. |  |  |  |  |  |  |  | |  |  |  |
|  | 2.4 | Understand the structure and function of the health system. |  |  |  |  |  |  |  | |  |  |  |
|  | 2.5 | Rational use of medical and health resources. |  |  |  |  |  |  |  | |  |  |  |
|  | 2.6 | Recognize the oral health for the individual and the important role of the health of the population, and actively participate in oral health education and health promotion. |  |  |  |  |  |  |  | |  |  |  |
|  | 2.7 | For the prevention and treatment of oral diseases |  |  |  |  |  |  |  | |  |  |  |
|  | 2.8 | Objectively assess the short-term and long-term effects of oral health strategies. |  |  |  |  |  |  |  | |  |  |  |
| **Primary indicator** |  | Second index | importance  （1-10） | feasibility  （1-10） | Sensitivity  （1-10） | Familiarity:  5 = Very familiar  4 = More familiar  3 = General  2 = Less familiar  1 = Unfamiliar | Judgment basis and degree of influence (degree of influence divided into three levels 3 = large, 2 = medium, 1 = small) | | | | | modify |  |
|  |  |  |  |  |  |  | Working Experience | Theoretical analysis | Peer understanding | | Intuition |  |  |
|  | 2.9 | Assess the patient's oral disease or risk factors for injury. |  |  |  |  |  |  |  | |  |  |  |
| Information Collection and Management Capability | 3.1 | Use different databases and other means to retrieve, collect, and analyze relevant medical information. |  |  |  |  |  |  |  | |  |  |  |
|  | 3.2 | Can use information technology effectively to communicate with doctors, nurses and mechanics, and educate patients with health knowledge. |  |  |  |  |  |  |  | |  |  |  |
|  | 3.3 | Reasonably control the patient's medical expenses. |  |  |  |  |  |  |  | |  |  |  |
|  | 3.4 | Effectively arrange their own work and do a good job of career planning. |  |  |  |  |  |  |  | |  |  |  |
| **Primary indicator** |  | Second index | importance  （1-10） | feasibility  （1-10） | Sensitivity  （1-10） | Familiarity:  5 = Very familiar  4 = More familiar  3 = General  2 = Less familiar  1 = Unfamiliar | Judgment basis and degree of influence (degree of influence divided into three levels 3 = large, 2 = medium, 1 = small) | | | | | modify |  |
|  |  |  |  |  |  |  | Working Experience | Theoretical analysis | Peer understanding | | Intuition |  |  |
| Information Collection and Management Capability | 3.5 | Can be well self-managed, and deal with their own activities reasonably. |  |  |  |  |  |  |  | |  |  |  |
|  | 3.6 | Continuously improve organizational coherence and leadership in medical practice.。 |  |  |  |  |  |  |  | |  |  |  |
|  | 3.7 | Have a certain professional foreign language skills. |  |  |  |  |  |  |  | |  |  |  |
|  | 3.8 | Keep an accurate, consistent, and clear patient management record, including referral, commission or transfer of records. |  |  |  |  |  |  |  | |  |  |  |
|  | 3.9 | Reasonable and effective management of patients. |  |  |  |  |  |  |  | |  |  |  |
|  | 3.10 | Can use modern information technology to conduct a reasonable publicize of the self. |  |  |  |  |  |  |  | |  |  |  |
| **Primary indicator** |  | Second index | importance  （1-10） | feasibility  （1-10） | Sensitivity  （1-10） | Familiarity:  5 = Very familiar  4 = More familiar  3 = General  2 = Less familiar  1 = Unfamiliar | Judgment basis and degree of influence (degree of influence divided into three levels 3 = large, 2 = medium, 1 = small) | | | | | modify |  |
|  |  |  |  |  |  |  | Working Experience | Theoretical analysis | Peer understanding | | Intuition |  |  |
| Medical Knowledge and Lifelong Learning Ability | 4.1 | Have basic biomedical knowledge. |  |  |  |  |  |  |  | |  |  |  |
|  | 4.2 | Knowledge of behavioral and social sciences, medical ethics and law. |  |  |  |  |  |  |  | |  |  |  |
|  | 4.3 | Master and apply the basic knowledge of clinical medicine. |  |  |  |  |  |  |  | |  |  |  |
|  | 4.4 | Practice concerns, including new materials and new technologies, including oral dynamic cutting-edge, constantly updated knowledge and professional skills |  |  |  |  |  |  |  | |  |  |  |
|  | 4.5 | Actively participate in continuing education. |  |  |  |  |  |  |  | |  |  |  |
| Interpersonal Communication Skills | 5.1 | Attentively listen, collect information related to synthesis and patient issues. |  |  |  |  |  |  |  | |  |  |  |
|  | 5.2 | Understand, trust and respect patients and their families. |  |  |  |  |  |  |  | |  |  |  |
| **Primary indicator** |  | Second index | importance  （1-10） | feasibility  （1-10） | Sensitivity  （1-10） | Familiarity:  5 = Very familiar  4 = More familiar  3 = General  2 = Less familiar  1 = Unfamiliar | Judgment basis and degree of influence (degree of influence divided into three levels 3 = large, 2 = medium, 1 = small) | | | | | modify |  |
|  |  |  |  |  |  |  | Working Experience | Theoretical analysis | Peer understanding | | Intuition |  |  |
| Interpersonal Communication Skills | 5.3 | Protection of patient privacy. |  |  |  |  |  |  |  | |  |  |  |
|  | 5.4 | To protect patients with the right to know, access to informed consent of patients. |  |  |  |  |  |  |  | |  |  |  |
|  | 5.5 | Properly deal with the ethical issues arising in the health care process. |  |  |  |  |  |  |  | |  |  |  |
|  | 5.6 | Appease the patient's anger and misunderstanding mood. |  |  |  |  |  |  |  | |  |  |  |
|  | 5.7 | Actively prevent and resolve doctor-patient conflicts. |  |  |  |  |  |  |  | |  |  |  |
|  | 5.8 | Euphemistically conveys negative news to patients. |  |  |  |  |  |  |  | |  |  |  |
|  | 5.9 | With the patients and their families to make clinical decisions. |  |  |  |  |  |  |  | |  |  |  |
| **Primary indicator** |  | Second index | importance  （1-10） | feasibility  （1-10） | Sensitivity  （1-10） | Familiarity:  5 = Very familiar  4 = More familiar  3 = General  2 = Less familiar  1 = Unfamiliar | Judgment basis and degree of influence (degree of influence divided into three levels 3 = large, 2 = medium, 1 = small) | | | | | modify |  |
|  |  |  |  |  |  |  | Working Experience | Theoretical analysis | Peer understanding | | Intuition |  |  |
| Interpersonal Communication Skills | 5.10 | Effective oral expression and transmission of information capabilities. |  |  |  |  |  |  |  | |  |  |  |
|  | 5.11 | Communicate effectively with patients, parents or guardians, employees, colleagues, other health professionals and the public. |  |  |  |  |  |  |  | |  |  |  |
| Teamwork | 6.1 | Be able to work with colleagues and respect their abilities and contributions. |  |  |  |  |  |  |  | |  |  |  |
|  | 6.2 | Develop a patient-care plan in a team-based manner. |  |  |  |  |  |  |  | |  |  |  |
|  | 6.3 | Caring for colleagues, willing to help colleagues. |  |  |  |  |  |  |  | |  |  |  |
|  | 6.4 | Understand the roles and responsibilities of others on the team. |  |  |  |  |  |  |  | |  |  |  |
| **Primary indicator** |  | Second index | importance  （1-10） | feasibility  （1-10） | Sensitivity  （1-10） | Familiarity:  5 = Very familiar  4 = More familiar  3 = General  2 = Less familiar  1 = Unfamiliar | judgment basis and degree of influence (degree of influence divided into three levels 3 = large, 2 = medium, 1 = small) | | | | | modify |  |
|  |  |  |  |  |  |  | Working Experience | Theoretical analysis | Peer understanding | | Intuition |  |  |
| Teamwork | 6.5 | Good coordination with team members to avoid conflict. |  |  |  |  |  |  |  | |  |  |  |
|  | 6.6 | Can establish good cooperative relationship with other teams. |  |  |  |  |  |  |  | |  |  |  |
|  | 6.7 | With the teachers to teach and the superior doctor's clinical treatment decisions of the implementation of the ability to well obey and perform a doctor's orders. |  |  |  |  |  |  |  | |  |  |  |
| Scientific Research Ability | 7.1 | Have critical thinking skills in professional activities and make appropriate medical decisions. |  |  |  |  |  |  |  | |  |  |  |
|  | 7.2 | Understand the complexity and uncertainty of health care activities. |  |  |  |  |  |  |  | |  |  |  |
| **Primary indicator** |  | Second index | importance  （1-10） | feasibility  （1-10） | Sensitivity  （1-10） | Familiarity:  5 = Very familiar  4 = More familiar  3 = General  2 = Less familiar  1 = Unfamiliar | judgment basis and degree of influence (degree of influence divided into three levels 3 = large, 2 = medium, 1 = small) | | | | | | modify | modify | modify | modify | | --- | --- | --- | --- | |  |
|  |  |  |  |  |  |  | Working Experience | Theoretical analysis | Peer understanding | | Intuition |  |  |
| Scientific Research Ability | 7.3 | Have the ability to read the literature, to carry out an academic literature review, and application and dissemination of knowledge. |  |  |  |  |  |  |  | |  |  |  |
|  | 7.4 | Can raise questions and assumptions, and consciously develop their own creative thinking and innovation. |  |  |  |  |  |  |  | |  |  |  |
|  | 7.5 | Actively participate in the research activities in this field. |  |  |  |  |  |  |  | |  |  |  |
|  | 7.6 | Write and publish research articles actively. |  |  |  |  |  |  |  | |  |  |  |
|  | 8.1 | Adhere to the principle during their career: serve the patient and care about the patient's health. |  |  |  |  |  |  |  | |  |  |  |
| **Primary indicator** |  | Second index | importance  （1-10） | feasibility  （1-10） | Sensitivity  （1-10） | Familiarity:  5 = Very familiar  4 = More familiar  3 = General  2 = Less familiar  1 = Unfamiliar | judgment basis and degree of influence (degree of influence divided into three levels 3 = large, 2 = medium, 1 = small) | | | | | modify |  |
|  |  |  |  |  |  |  | Working Experience | Theoretical analysis | Peer understanding | | Intuition |  |  |
| Core Values and Professional Qualities of Doctors | 8.2 | Cultivating core values, including altruism, pursuing excellence and indifferent to fame and fortune. |  |  |  |  |  |  |  | |  |  |  |
|  | 8.3 | Sincere and trustworthy, strong sense of responsibility, with a positive attitude and professionalism. |  |  |  |  |  |  |  | |  |  |  |
|  | 8.4 | With the idea of love and compassion, safeguard patients’ rights, privacy and interests. |  |  |  |  |  |  |  |  | |  |  |
|  | 8.5 | Show self-discipline and patient-centeredness in order to maximize achieving the interests of patients. |  |  |  |  |  |  |  |  | |  |  |
| **Primary indicator** |  | Second index | importance  （1-10） | feasibility  （1-10） | Sensitivity  （1-10） | Familiarity:  5 = Very familiar  4 = More familiar  3 = General  2 = Less familiar  1 = Unfamiliar | judgment basis and degree of influence (degree of influence divided into three levels 3 = large, 2 = medium, 1 = small) | | | | | modify |  |
|  |  |  |  |  |  |  | Working Experience | Theoretical analysis | Peer understanding | | Intuition |  |  |
| Core Values and Professional Qualities of Doctors | 8.6 | Have rigorous, meticulous, and keen insight. |  |  |  |  |  |  |  | |  |  |  |
|  | 8.7 | Physical and mental health, with patience and endurance, have a good psychological adjustment and compression capacity, maintain self-care ability. |  |  |  |  |  |  |  | |  |  |  |
|  | 8.8 | Fairly and reasonably use medical services resources. |  |  |  |  |  |  |  | |  |  |  |
|  | 8.9 | Have occupational health and occupational protection awareness, reduce the oral process of occupational hazards. |  |  |  |  |  |  |  | |  |  |  |
|  | 8.10 | Have a contingent capacity and response to emergency. |  |  |  |  |  |  |  | |  |  |  |
| **Primary indicator** |  | Second index | importance  （1-10） | feasibility  （1-10） | Sensitivity  （1-10） | Familiarity:  5 = Very familiar  4 = More familiar  3 = General  2 = Less familiar  1 = Unfamiliar | judgment basis and degree of influence (degree of influence divided into three levels 3 = large, 2 = medium, 1 = small) | | | | | modify |  |
|  |  |  |  |  |  |  | Working Experience | Theoretical analysis | Peer understanding | | Intuition |  |  |
| Core Values and Professional Qualities of Doctors | 8.11 | With the identity of the doctor's makeup. |  |  |  |  |  |  |  | |  |  |  |
|  | 8.12 | Ability to assess the clinical expertise of the individual, to recognize the limitations of the individual, and to know when to consult or seek advice. |  |  |  |  |  |  |  | |  |  |  |
|  | 8.13 | With the protection of peer awareness, have respect for peer review and treatment advice and recommendations. |  |  |  |  |  |  |  | |  |  |  |
|  | 8.14 | In the potential medical disputes have early warning consciousness |  |  |  |  |  |  |  | |  |  |  |
| modify |  | | | | | | | | | | | |  |

Note: Based on the feedback from the first round of experts, some indicators were deleted by statistical analysis and marked with green
