# Supplementary material for: Competency model for dentists in China: Results of a Delphi study
Source: PLoS One. 2018 Mar 22;13(3):e0194411. doi: 10.1371/journal.pone.0194411 (PMC5864007; doi:10.1371/journal.pone.0194411)
Supplement: S3 File — (DOC) [file pone.0194411.s003.doc]

**“5+1”阶段口腔执业医师准入岗位胜任力模型研究**

**专家咨询调查表(第一轮)**

**尊敬的专家:**您好!

感谢您在百忙之中参与专家咨询！为使您进一步了解我们的研究,请您先阅读本研究相关的内容及本次专家咨询的基本情况。

我们承担了国家医学考试中心委托的“口腔执业医师准入岗位胜任力模型研究”课题，本研究旨在通过专家咨询法及德尔菲法，汇总口腔医学专业**五年大学本科毕业，完成一年试用期准备参加口腔执业医师资格考试者（即“5+1”阶段）**应具备的能力，从而构建出胜任力指标体系，初步分为两级，一级指标为8个，即临床技能和医疗服务、疾病预防和健康促进、信息与管理能力、医学知识与终身学习能力、人际沟通能力、团队合作能力、科学研究能力、核心价值观与医生职业素养，在一级指标下进一步开发二级指标并制定权重系数，形成科学合理、具有可操作的指标体系，构建适合我国国情的口腔医师岗位胜任力模型。旨在为今后研究探索在口腔类别医师资格考试中引入“岗位胜任力”理念提供研究依据，并且为口腔医师的培养、选聘、培训和考核过程提供更为科学化和量化的依据。

为节约时间并保证研究进度,我们采用邮件方式对您进**两轮专家咨询**，请您在百忙之中逐项填写问卷、避免遗漏,**您的选择和打分对我们来说至关重要，请在一周内填完发至kqyssrl@sina.com**。如果您对问卷有疑问,请拨打电话或发送E-mail咨询。对您填写的调查问卷我们将会严格保密，衷心感谢您在百忙之中对本研究的支持!

联系人:

孟 开 13261365511 郑东翔 13681036988

国家医学考试中心“口腔执业医师准入岗位胜任力模型”课题组

首都医科大学附属北京口腔医院

二〇一六年一月

**专家基本情况调查**

1. 您的姓名：___ __________；工作单位：___ __________；

联系电话：_ ___________；电子邮箱：___ __________；

1. 您的工作职务: ___ ______；专业技术职称：___ __________。
2. 您的文化程度：（请在对应选项前划“√”）

①高中、中专 ②大专 ③本科 ④硕士 ⑤博士及以上

4、您的工作年限:

①≤5年 ②6—10年 ③11—15年 ④16—20年 ⑤>20年

5、目前主要从事的专业领域（可多选）：

①牙体牙髓科 ②牙周科 ③黏膜科 ④口腔颌面外科 ⑤口腔修复科

⑥口腔正畸科 ⑦儿童口腔科 ⑧口腔预防科 ⑨口腔种植科 ⑩口腔综合科

护理 其他___________ _

6、您目前主要担任的工作（可多选）：

①医疗工作 ②护理工作 ③教学工作 ④科研工作 ⑤医院管理工作 ⑥其他___________

**指标选择标准的说明**

1. 指标的重要性：是指指标的重要程度，重要性越高，说明该指标越重要，1分为最不重要，10分为最重要；
2. 指标的可行性：是指在实际评价工作中，获取该指标的难易程度和成本代价。指标越容易获得，该指标的可行性越高，1分最不具可行性，10分最具可行性；
3. 指标的敏感性：是指指标是否能较好地反映出 “5+1”阶段口腔医学专业医学生应具备的能力，如果某指标的敏感性强，则说明这个指标的改进可以较大程度增强对口腔医师胜任力的反映程度。选取1-10分十个分值，表示指标的敏感性依次增强；
4. 本次研究设计的指标体系较多，您认为哪个指标不重要或不易获得（不可行），请于指标后方划“**×**”，若要增加指标，则填写在下方建议栏中，并打分；
5. 您对指标的判断受“1理论分析、2实践经验、3同行了解、4直觉”四方面因素影响，影响程度分为大（3分）、中（2分）、小（1分）三个级别，请您根据您对每个指标的判断根据，在指标后选择相应的影响程度；
6. 您对指标的了解程度分为“5非常熟悉、4较熟悉、3一般、2较不熟悉、1很不熟悉”，请您根据实际情况，将相应序号填写在表格中。

| **表1：一级指标体系评价表** | | | | | | | | | | |  |
| --- | --- | --- | --- | --- | --- | --- | --- | --- | --- | --- | --- |
| 编号 | 一级指标 | 重要性  （1-10分） | 可行性  （1-10分） | 敏感性  （1-10分） | 熟悉程度:（**请填写数字，单选**）  5=很熟悉 4=较熟悉 3=—般熟悉 2=较不熟悉 1=很不熟悉 | 判断依据及影响程度（影响程度分为大、中、小三个层次，3=大，2=中，1=小，以下**请填写数字**） | | | | 修改意见（删除、合并、重新表述一级指标） | |
|  |  |  |  |  |  | 理论分析 | 工作经验 | 国内外同行了解 | 直觉 |  |  |
| 1 | 临床技能和医疗服务 |  |  |  |  |  |  |  |  |  | |
| 2 | 疾病预防和健康促进 |  |  |  |  |  |  |  |  |  | |
| 3 | 信息与管理能力 |  |  |  |  |  |  |  |  |  | |
| 4 | 医学知识与终身学习能力 |  |  |  |  |  |  |  |  |  | |
| 5 | 人际沟通能力 |  |  |  |  |  |  |  |  |  | |
| 6 | 团队合作能力 |  |  |  |  |  |  |  |  |  | |
| 7 | 科学研究能力 |  |  |  |  |  |  |  |  |  | |
| 8 | 核心价值观与医生职业素养 |  |  |  |  |  |  |  |  |  | |
| 修改建议： | | | | | | | | | | | |

**表2：二级指标体系评价表**

| 一级指标 | 编号 | 二级指标 | | 重要性  （1-10分） | | 可行性（1-10分） | 敏感性（1-10分） | 熟悉程度:（**请填写数字，单选**）  5=很熟悉 4=较熟悉 3=—般熟悉 2=较不熟悉 1=很不熟悉 | 判断依据及影响程度（影响程度分为大、中、中三个层次，3=大，2=中，1=小，以下**请填写数字**） | | | | | 修改意见（删除、合并、重新表述二级指标） | |  | |
| --- | --- | --- | --- | --- | --- | --- | --- | --- | --- | --- | --- | --- | --- | --- | --- | --- | --- |
|  |  |  |  |  |  |  |  |  | 理论分析 | | 工作经验 | 国内外同行了解 | 直觉 |  |  |  | |
| 1. 临床技能和医疗服务 | 1.1 | 完整准确的采集重要病史。 | |  | |  |  |  |  | |  |  |  |  | |  | |
|  | 1.2 | 比较规范地进行口腔相关体格检查。 | |  | |  |  |  |  | |  |  |  |  |  |  | |
|  | 1.3 | 能正确的选择辅助检查项目。 | |  | |  |  |  |  | |  |  |  |  |  |  | |
|  | 1.4 | 向上级医生规范的口头报告临床遇到的问题并能分析解释。 | |  | |  |  |  |  | |  |  |  |  |  |  | |
|  | 1.5 | 运用循证医学的方法做出医疗决策，采用合理的诊断和治疗计划。 | |  | |  |  |  |  | |  |  |  |  |  |  | |
|  | 1.6 | 能识别并积极参与对一般、急、重、危患者的现场治疗。 | |  | |  |  |  |  | |  |  |  |  |  |  | |
|  | 1.7 | 独立接诊能力。 | |  | |  |  |  |  | |  |  |  |  |  |  | |
|  | 1.8 | 多专业的综合分析能力。 | |  | |  |  |  |  | |  |  |  |  |  |  | |
|  | 1.9 | 将理论知识和临床实践相结合的能力。 | |  | |  |  |  |  | |  |  |  |  |  |  | |
|  | 1.10 | 正确使用常用器械、耗材、设备并能够熟练规范地进行基本的口腔治疗操作 。 | |  | |  |  |  |  | |  |  |  |  |  |  | |
|  | 1.11 | 规范的病历书写能力。 | |  | |  |  |  |  | |  |  |  |  |  |  | |
|  | 1.12 | 完成特定工作量的能力。 | |  | |  |  |  |  | |  |  |  |  |  |  | |
|  | 1.13 | 对疑难病例具有一定的独立分析能力。 | |  | |  |  |  |  | |  |  |  |  |  |  | |
|  | 1.14 | 对主流技术的掌握能力。 | |  | |  |  |  |  | |  |  |  |  |  |  | |
|  | 1.15 | 在口腔治疗过程中，考虑患者需求，解释病因、诊断、治疗结果、风险、利益以及不同治疗方案的预期效果，把握口腔诊疗的整体目标。 | |  | |  |  |  |  | |  |  |  |  |  |  | |
| 一级指标 | 编号 | 二级指标 | | 重要性  （1-10分） | | 可行性（1-10分） | 敏感性（1-10分） | 熟悉程度:（**请填写数字，单选**）  5=很熟悉 4=较熟悉 3=—般熟悉 2=较不熟悉 1=很不熟悉 | 判断依据及影响程度（影响程度分为大、中、中三个层次，3=大，2=中，1=小，以下**请填写数字**） | | | | | 修改意见（删除、合并、重新表述二级指标） | |  | |
|  |  |  |  |  |  |  |  |  | 理论分析 | | 工作经验 | 国内外同行了解 | 直觉 |  |  | | |
| 1. 临床技能和医疗服务 | 1.16 | 将专业术语转化成患者容易理解的语言为患者进行病情解释,提出并讨论治疗计划、费用预算、时间要求和患者应该承担的责任。 | |  | |  |  |  |  | |  |  |  |  | |  | |
|  | 1.17 | 掌握口腔手术局部麻醉并且治疗相关的并发症。 | |  | |  |  |  |  | |  |  |  |  |  |  | |
|  | 1.18 | 在口腔操作中明确使用药物的适应证和禁忌证，并且正确书写用于口腔治疗的药物处方。 | |  | |  |  |  |  | |  |  |  |  |  |  | |
|  | 1.19 | 能够鉴别患者口腔疾病的心理和社会因素，并恰当的处理心理与行为因素对口腔健康的不利作用。 | |  | |  |  |  |  | |  |  |  |  |  |  | |
| 2. 疾病预防和健康促进 | 2.1 | 发现和及时按规定上报法定传染病。 | |  | |  |  |  |  | |  |  |  |  | |  | |
|  | 2.2 | 为口腔疾病提供防治措施。 | |  | |  |  |  |  | |  |  |  |  | |  | |
|  | 2.3 | 了解自己的职责，与卫生系统管理人员合作。 | |  | |  |  |  |  | |  |  |  |  |  |  | |
|  | 2.4 | 了解医疗卫生体制的结构和功能。 | |  | |  |  |  |  | |  |  |  |  |  |  | |
|  | 2.5 | 合理利用医疗卫生资源。 | |  | |  |  |  |  | |  |  |  |  |  |  | |
|  | 2.6 | 认识到口腔健康对于个体及人群健康的重要作用，积极参与口腔健康教育与健康促进。 | |  | |  |  |  |  | |  |  |  |  |  |  | |
|  | 2.7 | 通过遵循当前的感染控制指导方针防止传染病的传播。 | |  | |  |  |  |  | |  |  |  |  |  |  | |
|  | 2.8 | 客观地评估口腔健康策略的短期和长期的效果。 | |  | |  |  |  |  | |  |  |  |  |  |  | |
|  | 2.9 | 评估患者的口腔疾病或损伤的危险因素。 | |  | |  |  |  |  | |  |  |  |  |  |  | |
| 3. 信息与管理能力 | 3.1 | 利用不同数据库等途径检索、收集、分析有关医学信息。 | |  | |  |  |  |  | |  |  |  |  | |  | |
| 一级指标 | 编号 | 二级指标 | | 重要性  （1-10分） | | 可行性（1-10分） | 敏感性（1-10分） | 熟悉程度:（**请填写数字，单选**）  5=很熟悉 4=较熟悉 3=—般熟悉 2=较不熟悉 1=很不熟悉 | 判断依据及影响程度（影响程度分为大、中、中三个层次，3=大，2=中，1=小，以下**请填写数字**） | | | | | 修改意见（删除、合并、重新表述二级指标） | |  | |
|  |  |  |  |  |  |  |  |  | 理论分析 | | 工作经验 | 国内外同行了解 | 直觉 |  |  |  | |
| 3. 信息与管理能力 | 3.2 | 有效利用信息技术进行医护技交流与患者健康教育。 | |  | |  |  |  |  | |  |  |  |  | |  | |
|  | 3.3 | 合理控制患者医疗费用。 | |  | |  |  |  |  | |  |  |  |  |  |  | |
|  | 3.4 | 有效安排自己的工作和职业生涯规划。 | |  | |  |  |  |  | |  |  |  |  |  |  | |
|  | 3.5 | 具备自我管理能力,能够有计划地处理自己的活动。 | |  | |  |  |  |  | |  |  |  |  |  |  | |
|  | 3.6 | 在医疗实践中不断提高组织协调和领导力。 | |  | |  |  |  |  | |  |  |  |  |  |  | |
|  | 3.7 | 具备一定的专业外语能力。 | |  | |  |  |  |  | |  |  |  |  |  |  | |
|  | 3.8 | 保留一份准确、一致、清晰的病人管理记录，包括转诊，委托或移交记录。 | |  | |  |  |  |  | |  |  |  |  |  |  | |
|  | 3.9 | 对病人进行合理有效的管理。 | |  | |  |  |  |  | |  |  |  |  |  |  | |
|  | 3.10 | 能够运用现代化信息科技技术对自我进行合理宣传。 | |  | |  |  |  |  | |  |  |  |  |  |  | |
| 4. 医学知识与终身学习能力 | 4.1 | 具备生物医学基础知识。 | |  | |  |  |  |  | |  |  |  |  | |  | |
|  | 4.2 | 具备行为和社会科学、医学伦理学及法学知识。 | |  | |  |  |  |  | |  |  |  |  |  |  | |
|  | 4.3 | 掌握与应用临床医学基本知识。 | |  | |  |  |  |  | |  |  |  |  |  |  | |
|  | 4.4 | 执业实践中关注包括新材料和新技术在内的口腔医学前沿动态，不断更新知识和专业技能。 | |  | |  |  |  |  | |  |  |  |  |  |  | |
|  | 4.5 | 积极参加继续教育。 | |  | |  |  |  |  | |  |  |  |  |  |  | |
| 5. 人际沟通能力 | 5.1 | 注意倾听、收集与综合和患者问题有关的信息。 | |  | |  |  |  |  | |  |  |  |  | |  | |
|  | 5.2 | 理解、信任并尊重患者及其家属。 | |  | |  |  |  |  | |  |  |  |  |  |  | |
|  | 5.3 | 保护患者隐私。 | |  | |  |  |  |  | |  |  |  |  |  |  | |
|  | 5.4 | 维护患者知情权，获得患者的知情同意。 | |  | |  |  |  |  | |  |  |  |  |  |  | |
| 一级指标 | 编号 | 二级指标 | | 重要性  （1-10分） | | 可行性（1-10分） | 敏感性（1-10分） | 熟悉程度:（**请填写数字，单选**）  5=很熟悉 4=较熟悉 3=—般熟悉 2=较不熟悉 1=很不熟悉 | 判断依据及影响程度（影响程度分为大、中、中三个层次，3=大，2=中，1=小，以下**请填写数字**） | | | | | 修改意见（删除、合并、重新表述二级指标） | |  | |
|  |  |  |  |  |  |  |  |  | 理论分析 | | 工作经验 | 国内外同行了解 | 直觉 |  |  |  | |
| 5. 人际沟通能力 | 5.5 | 妥善应对在医护过程中产生的伦理问题。 | |  | |  |  |  |  | |  |  |  |  | |  | |
|  | 5.6 | 安抚患者的愤怒和误解的情绪。 | |  | |  |  |  |  | |  |  |  |  |  |  | |
|  | 5.7 | 积极预防和化解医患矛盾。 | |  | |  |  |  |  | |  |  |  |  |  |  | |
|  | 5.8 | 委婉地向患者传达负面消息。 | |  | |  |  |  |  | |  |  |  |  |  |  | |
|  | 5.9 | 与患者和家属共同作出临床决策。 | |  | |  |  |  |  | |  |  |  |  |  |  | |
|  | 5.10 | 有效口头表达和传递信息能力。 | |  | |  |  |  |  | |  |  |  |  |  |  | |
|  | 5.11 | 与患者、父母或监护人、员工、同事、其他卫生专业人员以及公众进行有效沟通。 | |  | |  |  |  |  | |  |  |  |  |  |  | |
| 6. 团队合作能力 | 6.1 | 必须和同事合作，尊重他们的能力和贡献。 | |  | |  |  |  |  | |  |  |  |  | |  | |
|  | 6.2 | 以团队合作的方式制订患者的诊疗计划。 | |  | |  |  |  |  | |  |  |  |  |  |  | |
|  | 6.3 | 关心和乐于帮助同事。 | |  | |  |  |  |  | |  |  |  |  |  |  | |
|  | 6.4 | 了解团队中其他人的角色和职责。 | |  | |  |  |  |  | |  |  |  |  |  |  | |
|  | 6.5 | | 善于协调与团队成员关系，避免发生冲突。 | |  |  |  |  | |  |  |  |  |  |  |  | |
|  | 6.6 | 能与其他团队建立良好的合作关系。 | |  | |  |  |  |  | |  |  |  |  |  |  | |
|  | 6.7 | 具备对带教老师和上级医生的临床治疗决策的执行力，能够很好地服从并执行上级医生的医嘱。 | |  | |  |  |  |  | |  |  |  |  | |  | |
|  | 7.1 | 在职业活动中具备一定的批判性思维能力，并恰当地做出医疗决策。 | |  | |  |  |  |  | |  |  |  |  | |  | |
|  | 7.2 | 理解医疗活动的复杂性和不确定性。 | |  | |  |  |  |  | |  |  |  |  | |  | |
| 一级指标 | 编号 | 二级指标 | | 重要性  （1-10分） | | 可行性（1-10分） | 敏感性（1-10分） | 熟悉程度:（**请填写数字，单选**）  5=很熟悉 4=较熟悉 3=—般熟悉 2=较不熟悉 1=很不熟悉 | 判断依据及影响程度（影响程度分为大、中、中三个层次，3=大，2=中，1=小，以下**请填写数字**） | | | | | 修改意见（删除、合并、重新表述二级指标） | |  |  |
|  |  |  |  |  |  |  |  |  | 理论分析 | | 工作经验 | 国内外同行了解 | 直觉 |  |  |  | |
| 7. 科学研究能力 | 7.3 | 具备文献的阅读能力，能进行学术文献综述，并应用和传播知识。 | |  | |  |  |  |  | |  |  |  |  | |  | |
|  | 7.4 | 能提出问题和假设，培养创造性思维和创新能力。 | |  | |  |  |  |  | |  |  |  |  | |  | |
|  | 7.5 | 积极参加本专业领域的科研活动。 | |  | |  |  |  |  | |  |  |  |  |  |  | |
|  | 7.6 | 积极撰写并发表科研文章。 | |  | |  |  |  |  | |  |  |  |  |  |  | |
| 8. 核心价值观与医生职业素养 | 8.1 | 在职业生涯中坚持一切为人民健康服务的宗旨。 | |  | |  |  |  |  | |  |  |  |  | |  | |
|  | 8.2 | 培养核心价值观包括利他主义、追求卓越和淡泊名利。 | |  | |  |  |  |  | |  |  |  |  |  |  | |
|  | 8.3 | 真诚守信，责任心强，具有积极的工作态度和敬业精神。 | |  | |  |  |  |  | |  |  |  |  |  |  | |
|  | 8.4 | 具有爱伤观念和同情心，维护患者权利、隐私和利益。 | |  | |  |  |  |  | |  |  |  |  | |  | |
|  | 8.5 | 能够行业自律，以患者为中心，实现患者利益最大化。 | |  | |  |  |  |  | |  |  |  |  | |  | |
|  | 8.6 | 具备严谨、细致、敏锐的洞察力。 | |  | |  |  |  |  | |  |  |  |  | |  | |
|  | 8.7 | 身心健康，具备耐心和耐力，具备良好的心理调适和抗压能力，保持自我保健能力。 | |  | |  |  |  |  | |  |  |  |  | |  | |
|  | 8.8 | 公平而合理地运用各种医疗服务资源。 | |  | |  |  |  |  | |  |  |  |  | |  | |
|  | 8.9 | 具备职业健康和职业防护意识,减少口腔操作过程中的职业危害。 | |  | |  |  |  |  | |  |  |  |  | |  | |
| 一级指标 | 编号 | 二级指标 | | 重要性  （1-10分） | | 可行性（1-10分） | 敏感性（1-10分） | 熟悉程度:（**请填写数字，单选**）  5=很熟悉 4=较熟悉 3=—般熟悉 2=较不熟悉 1=很不熟悉 | 判断依据及影响程度（影响程度分为大、中、中三个层次，3=大，2=中，1=小，以下**请填写数字**） | | | | | 修改意见（删除、合并、重新表述二级指标） | |  |  |
|  |  |  | |  |  |  |  |  | 理论分析 | | 工作经验 | 国内外同行了解 | 直觉 |  | |  | |
| 8. 核心价值观与医生职业素养 | 8.10 | 对突发事件的应变能力和反应能力。 | |  | |  |  |  |  | |  |  |  |  | |  | |
|  | 8.11 | 具有符合医生身份的仪表妆容。 | |  | |  |  |  |  | |  |  |  |  | |  | |
|  | 8.12 | 能对自身临床专业能力进行正确评估，承认个人的局限性以及知道何时适当地咨询或寻求建议。 | |  | |  |  |  |  | |  |  |  |  | |  | |
|  | 8.13 | 具备同行间的保护意识，尊重同行的诊疗意见和建议。 | |  | |  |  |  |  | |  |  |  |  | |  | |
|  | 8.14 | 对潜在的医疗纠纷有预警意识。 | |  | |  |  |  |  | |  |  |  |  | |  | |
| 修改建议： |  | | | | | | | | | | | | | | |  | |
|  |  |  |  |  |  |  |  |  |  |  |  |  |  |  |  |  |  |
