# Supplementary material for: Competency model for dentists in China: Results of a Delphi study
Source: PLoS One. 2018 Mar 22;13(3):e0194411. doi: 10.1371/journal.pone.0194411 (PMC5864007; doi:10.1371/journal.pone.0194411)
Supplement: S4 File — (DOC) [file pone.0194411.s004.doc]

**“5+1”阶段口腔执业医师准入岗位胜任力模型研究**

**专家咨询调查问卷(第二轮)**

**尊敬的专家:**您好!

感谢您在第一轮专家咨询调查中给予的大力支持！我们根据各位专家对指标体系的评判和建议，对指标进行了删减和修改。

我们承担了国家医学考试中心委托的“口腔执业医师准入岗位胜任力模型研究”课题，本研究旨在通过专家咨询法及德尔菲法，汇总口腔医学专业**五年大学本科毕业，完成一年试用期准备参加口腔执业医师资格考试者（即“5+1”阶段）**应具备的能力，从而构建出胜任力指标体系，初步分为两级，一级指标为8个，即临床技能和医疗服务、疾病预防和健康促进、信息与管理能力、医学知识与终身学习能力、人际沟通能力、团队合作能力、科学研究能力、核心价值观与医生职业素养，在一级指标下进一步开发二级指标并制定权重系数，形成科学合理、具有可操作的指标体系，构建适合我国国情的口腔医师岗位胜任力模型。旨在为今后研究探索在口腔类别医师资格考试中引入“岗位胜任力”理念提供研究依据，并且为口腔医师的培养、选聘、培训和考核过程提供更为科学化和量化的依据。

为节约时间并保证研究进度,我们采用邮件方式对您进行**第二轮专家咨询**，请您在百忙之中逐项填写问卷、避免遗漏,**您的选择和打分对我们来说至关重要，请在一周内填完发至kqyssrl@sina.com**。如果您对问卷有疑问,请拨打电话或发送E-mail咨询。对您填写的调查问卷我们将会严格保密，衷心感谢您在百忙之中对本研究的支持!

联系人:

孟 开 13261365511 郑东翔 13681036988

国家医学考试中心“口腔执业医师准入岗位胜任力模型”课题组

首都医科大学附属北京口腔医院

二〇一六年四月

**指标选择标准的说明**

1. 指标的重要性：是指指标的重要程度，重要性越高，说明该指标越重要，1分为最不重要，10分为最重要；
2. 指标的可行性：是指在实际评价工作中，获取该指标的难易程度和成本代价。指标越容易获得，该指标的可行性越高，1分最不具可行性，10分最具可行性；
3. 指标的敏感性：是指指标是否能较好地反映出 “5+1”阶段口腔医学专业医学生应具备的能力，1分为最不敏感，10分为最敏感；
4. 本次研究设计的指标体系较多，您认为哪个指标不重要或不易获得（不可行），请于指标后方划“**×**”，若要增加指标，则填写在下方建议栏中，并打分；
5. 您对指标的判断受“1理论分析、2实践经验、3从同行了解、4直觉”四方面因素影响，影响程度分为大（3分）、中（2分）、小（1分）三个级别，请您根据您对每个指标的判断根据，在指标后选择相应的影响程度；
6. 您对指标的了解程度分为“5非常熟悉、4较熟悉、3一般、2较不熟悉、1很不熟悉”，请您根据实际情况，将相应序号填写在表格中；

**一级指标的权重的计算**

（1）计算方法：第二轮专家咨询采用**层次分析法**计算一级指标权重

（2）指标相对重要程度说明：

**表1 重要性程度**说明

| 重要性程度 | 含义 | 重要性程度 | 含义 |
| --- | --- | --- | --- |
| 1 | A指标和B指标相比，具有同等重要性； | 1 | A指标和B指标相比，具有同等重要性； |
| 3 | A指标和B指标相比，前者比后者稍重要； | 1/3 | A指标和B指标相比，后者比前者稍重要； |
| 5 | A指标和B指标相比，前者比后者明显重要； | 1/5 | A指标和B指标相比，后者比前者明显重要； |
| 7 | A指标和B指标相比，前者比后者强烈重要； | 1/7 | A指标和B指标相比，后者比前者强烈重要； |
| 9 | A指标和B指标相比，前者比后者极端重要； | 1/9 | A指标和B指标相比，后者比前者极端重要； |
| 2、4、6、8 | 表示上述相邻两个判断的折中值 | 1/2、1/4、1/6、1/8 | 表示上述相邻两个判断的折中值 |

**表2 一级指标重要程度调查表（A与B相比）**

| **B A** | 临床技能和医疗服务（B） | 疾病预防和健康促进（B） | 信息与管理能力（B） | 医学知识与终身学习能力（B） | 人际沟通能力（B） | 团队合作能力（B） | 科学研究能力（B） | 核心价值观与医生职业素养（B） |
| --- | --- | --- | --- | --- | --- | --- | --- | --- |
| 临床技能和医疗服务（A） | 1 |  |  |  |  |  |  |  |
| 疾病预防和健康促进（A） |  | 1 |  |  |  |  |  |  |
| 信息与管理能力（A） |  |  | 1 |  |  |  |  |  |
| 医学知识与终身学习能力（A） |  |  |  | 1 |  |  |  |  |
| 人际沟通能力（A） |  |  |  |  | 1 |  |  |  |
| 团队合作能力（A） |  |  |  |  |  | 1 |  |  |
| 科学研究能力（A） |  |  |  |  |  |  | 1 |  |
| 核心价值观与医生职业素养（A） |  |  |  |  |  |  |  | 1 |

**注：画有斜线和“1”的表格不用填写**

**表3: 一级指标体系评价表**

| 编号 | 一级指标 | 熟悉程度:（**请填写数字，单选**）  5=很熟悉 4=较熟悉 3=—般熟悉 2=较不熟悉 1=很不熟悉 | 判断依据及影响程度（影响程度分为大、中、小三个层次，3=大，2=中，1=小，以下**请填写数字**） | | | | 修改意见（删除、合并、重新表述一级指标） |
| --- | --- | --- | --- | --- | --- | --- | --- |
|  |  |  | 理论分析 | 工作经验 | 从国内外同行了解 | 直觉 |  |
| 1 | 临床技能和医疗服务 |  |  |  |  |  |  |
| 2 | 疾病预防和健康促进 |  |  |  |  |  |  |
| 3 | 信息与管理能力 |  |  |  |  |  |  |
| 4 | 医学知识与终身学习能力 |  |  |  |  |  |  |
| 5 | 人际沟通能力 |  |  |  |  |  |  |
| 6 | 团队合作能力 |  |  |  |  |  |  |
| 7 | 科学研究能力 |  |  |  |  |  |  |
| 8 | 核心价值观与医生职业素养 |  |  |  |  |  |  |

**表4：二级指标体系评价表**

| 一级指标 | 编号 | 二级指标 | | 重要性  （1-10分） | | 可行性（1-10分） | 敏感性（1-10分） | 熟悉程度:（**请填写数字，单选**）  5=很熟悉 4=较熟悉 3=—般熟悉 2=较不熟悉 1=很不熟悉 | 判断依据及影响程度（影响程度分为大、中、中三个层次，3=大，2=中，1=小，以下**请填写数字**） | | | | | 修改意见（删除、合并、重新表述二级指标） | |  | |
| --- | --- | --- | --- | --- | --- | --- | --- | --- | --- | --- | --- | --- | --- | --- | --- | --- | --- |
|  |  |  |  |  |  |  |  |  | 理论分析 | | 工作经验 | 从国内外同行了解 | 直觉 |  |  |  | |
| 1. 临床技能和医疗服务 | 1.1 | 完整准确的采集重要病史。 | |  | |  |  |  |  | |  |  |  |  | |  | |
|  | 1.2 | 比较规范地进行口腔相关体格检查。 | |  | |  |  |  |  | |  |  |  |  |  |  | |
|  | 1.3 | 能正确的选择辅助检查项目。 | |  | |  |  |  |  | |  |  |  |  |  |  | |
|  | 1.4 | 向上级医生规范的口头报告临床遇到的问题并能分析解释。 | |  | |  |  |  |  | |  |  |  |  |  |  | |
|  | 1.5 | 运用循证医学的方法做出医疗决策，采用合理的诊断和治疗计划。 | |  | |  |  |  |  | |  |  |  |  |  |  | |
|  | 1.6 | 能识别并积极参与对一般、急、重、危患者的现场治疗。 | |  | |  |  |  |  | |  |  |  |  |  |  | |
|  | 1.7 | 独立接诊能力。 | |  | |  |  |  |  | |  |  |  |  |  |  | |
|  | 1.8 | 多专业的综合分析能力。 | |  | |  |  |  |  | |  |  |  |  |  |  | |
|  | 1.9 | 将理论知识和临床实践相结合的能力。 | |  | |  |  |  |  | |  |  |  |  |  |  | |
|  | 1.10 | 正确使用常用器械、耗材、设备并能够熟练规范地进行基本的口腔治疗操作 。 | |  | |  |  |  |  | |  |  |  |  |  |  | |
|  | 1.11 | 规范的病历书写能力。 | |  | |  |  |  |  | |  |  |  |  |  |  | |
|  | 1.12 | 完成特定工作量的能力。 | |  | |  |  |  |  | |  |  |  |  |  |  | |
|  | 1.13 | 对疑难病例具有一定的独立分析能力。 | |  | |  |  |  |  | |  |  |  |  |  |  | |
|  | 1.14 | 对主流技术的掌握能力。 | |  | |  |  |  |  | |  |  |  |  |  |  | |
|  | 1.15 | 在口腔治疗过程中，考虑患者需求，解释病因、诊断、治疗结果、风险、利益以及不同治疗方案的预期效果，把握口腔诊疗的整体目标。 | |  | |  |  |  |  | |  |  |  |  |  |  | |
| 一级指标 | 编号 | 二级指标 | | 重要性  （1-10分） | | 可行性（1-10分） | 敏感性（1-10分） | 熟悉程度:（**请填写数字，单选**）  5=很熟悉 4=较熟悉 3=—般熟悉 2=较不熟悉 1=很不熟悉 | 判断依据及影响程度（影响程度分为大、中、中三个层次，3=大，2=中，1=小，以下**请填写数字**） | | | | | 修改意见（删除、合并、重新表述二级指标） | |  | |
|  |  |  |  |  |  |  |  |  | 理论分析 | | 工作经验 | 从国内外同行了解 | 直觉 |  |  | | |
| 1. 临床技能和医疗服务 | 1.16 | 将专业术语转化成患者容易理解的语言为患者进行病情解释,提出并讨论治疗计划、费用预算、时间要求和患者应该承担的责任。 | |  | |  |  |  |  | |  |  |  |  | |  | |
|  | 1.17 | 掌握口腔手术局部麻醉并且治疗相关的并发症。 | |  | |  |  |  |  | |  |  |  |  |  |  | |
|  | 1.18 | 在口腔操作中明确使用药物的适应证和禁忌证，并且正确书写用于口腔治疗的药物处方。 | |  | |  |  |  |  | |  |  |  |  |  |  | |
|  | 1.19 | 能够鉴别患者口腔疾病的心理和社会因素，并恰当的处理心理与行为因素对口腔健康的不利作用。 | |  | |  |  |  |  | |  |  |  |  |  |  | |
| 2. 疾病预防和健康促进 | 2.1 | 发现和及时按规定上报法定传染病。 | |  | |  |  |  |  | |  |  |  |  | |  | |
|  | 2.2 | 为口腔疾病提供防治措施。 | |  | |  |  |  |  | |  |  |  |  | |  | |
|  | 2.3 | 了解自己的职责，与卫生系统管理人员合作。 | |  | |  |  |  |  | |  |  |  |  |  |  | |
|  | 2.4 | 了解医疗卫生体制的结构和功能。 | |  | |  |  |  |  | |  |  |  |  |  |  | |
|  | 2.5 | 合理利用医疗卫生资源。 | |  | |  |  |  |  | |  |  |  |  |  |  | |
|  | 2.6 | 认识到口腔健康对于个体及人群健康的重要作用，积极参与口腔健康教育与健康促进。 | |  | |  |  |  |  | |  |  |  |  |  |  | |
|  | 2.7 | 通过遵循当前的感染控制指导方针防止传染病的传播。 | |  | |  |  |  |  | |  |  |  |  |  |  | |
|  | 2.8 | 客观地评估口腔健康策略的短期和长期的效果。 | |  | |  |  |  |  | |  |  |  |  |  |  | |
|  | 2.9 | 评估患者的口腔疾病或损伤的危险因素。 | |  | |  |  |  |  | |  |  |  |  |  |  | |
| 3. 信息与管理能力 | 3.1 | 利用不同数据库等途径检索、收集、分析有关医学信息。 | |  | |  |  |  |  | |  |  |  |  | |  | |
| 一级指标 | 编号 | 二级指标 | | 重要性  （1-10分） | | 可行性（1-10分） | 敏感性（1-10分） | 熟悉程度:（**请填写数字，单选**）  5=很熟悉 4=较熟悉 3=—般熟悉 2=较不熟悉 1=很不熟悉 | 判断依据及影响程度（影响程度分为大、中、中三个层次，3=大，2=中，1=小，以下**请填写数字**） | | | | | 修改意见（删除、合并、重新表述二级指标） | |  | |
|  |  |  |  |  |  |  |  |  | 理论分析 | | 工作经验 | 从国内外同行了解 | 直觉 |  |  |  | |
| 3. 信息与管理能力 | 3.2 | 有效利用信息技术进行医护技交流与患者健康教育。 | |  | |  |  |  |  | |  |  |  |  | |  | |
|  | 3.3 | 合理控制患者医疗费用。 | |  | |  |  |  |  | |  |  |  |  |  |  | |
|  | 3.4 | 有效安排自己的工作和职业生涯规划。 | |  | |  |  |  |  | |  |  |  |  |  |  | |
|  | 3.5 | 具备自我管理能力,能够有计划地处理自己的活动。 | |  | |  |  |  |  | |  |  |  |  |  |  | |
|  | 3.6 | 在医疗实践中不断提高组织协调和领导力。 | |  | |  |  |  |  | |  |  |  |  |  |  | |
|  | 3.7 | 具备一定的专业外语能力。 | |  | |  |  |  |  | |  |  |  |  |  |  | |
|  | 3.8 | 保留一份准确、一致、清晰的病人管理记录，包括转诊，委托或移交记录。 | |  | |  |  |  |  | |  |  |  |  |  |  | |
|  | 3.9 | 对病人进行合理有效的管理。 | |  | |  |  |  |  | |  |  |  |  |  |  | |
|  | 3.10 | 能够运用现代化信息科技技术对自我进行合理宣传。 | |  | |  |  |  |  | |  |  |  |  |  |  | |
| 4. 医学知识与终身学习能力 | 4.1 | 具备生物医学基础知识。 | |  | |  |  |  |  | |  |  |  |  | |  | |
|  | 4.2 | 具备行为和社会科学、医学伦理学及法学知识。 | |  | |  |  |  |  | |  |  |  |  |  |  | |
|  | 4.3 | 掌握与应用临床医学基本知识。 | |  | |  |  |  |  | |  |  |  |  |  |  | |
|  | 4.4 | 执业实践中关注包括新材料和新技术在内的口腔医学前沿动态，不断更新知识和专业技能。 | |  | |  |  |  |  | |  |  |  |  |  |  | |
|  | 4.5 | 积极参加继续教育。 | |  | |  |  |  |  | |  |  |  |  |  |  | |
| 5. 人际沟通能力 | 5.1 | 注意倾听、收集与综合和患者问题有关的信息。 | |  | |  |  |  |  | |  |  |  |  | |  | |
|  | 5.2 | 理解、信任并尊重患者及其家属。 | |  | |  |  |  |  | |  |  |  |  |  |  | |
|  | 5.3 | 保护患者隐私。 | |  | |  |  |  |  | |  |  |  |  |  |  | |
|  | 5.4 | 维护患者知情权，获得患者的知情同意。 | |  | |  |  |  |  | |  |  |  |  |  |  | |
| 一级指标 | 编号 | 二级指标 | | 重要性  （1-10分） | | 可行性（1-10分） | 敏感性（1-10分） | 熟悉程度:（**请填写数字，单选**）  5=很熟悉 4=较熟悉 3=—般熟悉 2=较不熟悉 1=很不熟悉 | 判断依据及影响程度（影响程度分为大、中、中三个层次，3=大，2=中，1=小，以下**请填写数字**） | | | | | 修改意见（删除、合并、重新表述二级指标） | |  | |
|  |  |  |  |  |  |  |  |  | 理论分析 | | 工作经验 | 从国内外同行了解 | 直觉 |  |  |  | |
| 5. 人际沟通能力 | 5.5 | 妥善应对在医护过程中产生的伦理问题。 | |  | |  |  |  |  | |  |  |  |  | |  | |
|  | 5.6 | 安抚患者的愤怒和误解的情绪。 | |  | |  |  |  |  | |  |  |  |  |  |  | |
|  | 5.7 | 积极预防和化解医患矛盾。 | |  | |  |  |  |  | |  |  |  |  |  |  | |
|  | 5.8 | 委婉地向患者传达负面消息。 | |  | |  |  |  |  | |  |  |  |  |  |  | |
|  | 5.9 | 与患者和家属共同做出临床决策。 | |  | |  |  |  |  | |  |  |  |  |  |  | |
|  | 5.10 | 有效口头表达和传递信息能力。 | |  | |  |  |  |  | |  |  |  |  |  |  | |
|  | 5.11 | 与患者、父母或监护人、员工、同事、其他卫生专业人员以及公众进行有效沟通。 | |  | |  |  |  |  | |  |  |  |  |  |  | |
| 6. 团队合作能力 | 6.1 | 必须和同事合作，尊重他们的能力和贡献。 | |  | |  |  |  |  | |  |  |  |  | |  | |
|  | 6.2 | 以团队合作的方式制订患者的诊疗计划。 | |  | |  |  |  |  | |  |  |  |  |  |  | |
|  | 6.3 | 关心和乐于帮助同事。 | |  | |  |  |  |  | |  |  |  |  |  |  | |
|  | 6.4 | 了解团队中其他人的角色和职责。 | |  | |  |  |  |  | |  |  |  |  |  |  | |
|  | 6.5 | | 善于协调与团队成员关系，避免发生冲突。 | |  |  |  |  | |  |  |  |  |  |  |  | |
|  | 6.6 | 能与其他团队建立良好的合作关系。 | |  | |  |  |  |  | |  |  |  |  |  |  | |
|  | 6.7 | 具备对带教老师和上级医生的临床治疗决策的执行力，能够很好地服从并执行上级医生的医嘱。 | |  | |  |  |  |  | |  |  |  |  | |  | |
|  | 7.1 | 在职业活动中具备一定的批判性思维能力，并恰当地做出医疗决策。 | |  | |  |  |  |  | |  |  |  |  | |  | |
|  | 7.2 | 理解医疗活动的复杂性和不确定性。 | |  | |  |  |  |  | |  |  |  |  | |  | |
| 一级指标 | 编号 | 二级指标 | | 重要性  （1-10分） | | 可行性（1-10分） | 敏感性（1-10分） | 熟悉程度:（**请填写数字，单选**）  5=很熟悉 4=较熟悉 3=—般熟悉 2=较不熟悉 1=很不熟悉 | 判断依据及影响程度（影响程度分为大、中、中三个层次，3=大，2=中，1=小，以下**请填写数字**） | | | | | 修改意见（删除、合并、重新表述二级指标） | |  |  |
|  |  |  |  |  |  |  |  |  | 理论分析 | | 工作经验 | 从国内外同行了解 | 直觉 |  |  |  | |
| 7. 科学研究能力 | 7.3 | 具备文献的阅读能力，能进行学术文献综述，并应用和传播知识。 | |  | |  |  |  |  | |  |  |  |  | |  | |
|  | 7.4 | 能提出问题和假设，培养创造性思维和创新能力。 | |  | |  |  |  |  | |  |  |  |  | |  | |
|  | 7.5 | 积极参加本专业领域的科研活动。 | |  | |  |  |  |  | |  |  |  |  |  |  | |
|  | 7.6 | 积极撰写并发表科研文章。 | |  | |  |  |  |  | |  |  |  |  |  |  | |
| 8. 核心价值观与医生职业素养 | 8.1 | 在职业生涯中坚持一切为人民健康服务的宗旨。 | |  | |  |  |  |  | |  |  |  |  | |  | |
|  | 8.2 | 培养核心价值观包括利他主义、追求卓越和淡泊名利。 | |  | |  |  |  |  | |  |  |  |  |  |  | |
|  | 8.3 | 真诚守信，责任心强，具有积极的工作态度和敬业精神。 | |  | |  |  |  |  | |  |  |  |  |  |  | |
|  | 8.4 | 具有爱伤观念和同情心，维护患者权利、隐私和利益。 | |  | |  |  |  |  | |  |  |  |  | |  | |
|  | 8.5 | 能够行业自律，以患者为中心，实现患者利益最大化。 | |  | |  |  |  |  | |  |  |  |  | |  | |
|  | 8.6 | 具备严谨、细致、敏锐的洞察力。 | |  | |  |  |  |  | |  |  |  |  | |  | |
|  | 8.7 | 身心健康，具备耐心和耐力，具备良好的心理调适和抗压能力，保持自我保健能力。 | |  | |  |  |  |  | |  |  |  |  | |  | |
|  | 8.8 | 公平而合理地运用各种医疗服务资源。 | |  | |  |  |  |  | |  |  |  |  | |  | |
|  | 8.9 | 具备职业健康和职业防护意识,减少口腔操作过程中的职业危害。 | |  | |  |  |  |  | |  |  |  |  | |  | |
| 一级指标 | 编号 | 二级指标 | | 重要性  （1-10分） | | 可行性（1-10分） | 敏感性（1-10分） | 熟悉程度:（**请填写数字，单选**）  5=很熟悉 4=较熟悉 3=—般熟悉 2=较不熟悉 1=很不熟悉 | 判断依据及影响程度（影响程度分为大、中、中三个层次，3=大，2=中，1=小，以下**请填写数字**） | | | | | 修改意见（删除、合并、重新表述二级指标） | |  |  |
|  |  |  | |  |  |  |  |  | 理论分析 | | 工作经验 | 从国内外同行了解 | 直觉 |  | |  | |
| 8. 核心价值观与医生职业素养 | 8.10 | 对突发事件的应变能力和反应能力。 | |  | |  |  |  |  | |  |  |  |  | |  | |
|  | 8.11 | 具有符合医生身份的仪表妆容。 | |  | |  |  |  |  | |  |  |  |  | |  | |
|  | 8.12 | 能对自身临床专业能力进行正确评估，承认个人的局限性以及知道何时适当地咨询或寻求建议。 | |  | |  |  |  |  | |  |  |  |  | |  | |
|  | 8.13 | 具备同行间的保护意识，尊重同行的诊疗意见和建议。 | |  | |  |  |  |  | |  |  |  |  | |  | |
|  | 8.14 | 对潜在的医疗纠纷有预警意识。 | |  | |  |  |  |  | |  |  |  |  | |  | |
| 修改建议： |  | | | | | | | | | | | | | | |  | |
|  |  |  |  |  |  |  |  |  |  |  |  |  |  |  |  |  |  |

**注:上表中,根据第一轮专家的反馈意见，经统计分析删除了部分指标,并用绿色标记**
